# Supplementary material for: sTREM-1 Predicts Disease Severity and Mortality in COVID-19 Patients: Involvement of Peripheral Blood Leukocytes and MMP-8 Activity
Source: Viruses. 2021 Dec 15;13(12):2521. doi: 10.3390/v13122521 (PMC8708887; doi:10.3390/v13122521)
Supplement: Supplementary file 1 [file viruses-13-02521-s001.zip › viruses-1425220-supplementary.pdf]

**Supplementary Information**

**Table S1.** Characterization of participants according to signs and symptoms of COVID-19.

| Participant's Classification | Symptoms, Signs, and Parameters |
|---|---|
| **Healthy Controls** | - Negative for SARS-Cov-2 nucleic acid<br>- No clinical signs |
| **Mild** | - Positive for SARS-Cov-2 nucleic acid and/or serological test<br>- With or without the following symptoms: diarrhea, cough, fever, headache, loss of taste (ageusia) / smell (anosmia), myalgia, nausea, and vomiting<br>- Oxygen saturation 94-99 % on room air |
| **Moderate** | - Positive for SARS-Cov-2 nucleic acid and/or serological test<br>- Manifestation of mild disease symptoms including dyspnea<br>- Oxygen saturation ≥ 93% on room air and $PaO_2/FiO_2$ 250-300 mmHg<br>- Do not need invasive ventilation: nasal catheter (oxygen 2-4 L/min) or oxygen reservoir (oxygen 4-12 L/min) |
| **Severe** | - Positive for SARS-Cov-2 nucleic acid and/or serological test<br>- Possible Admission to intensive-care units<br>- Severe respiratory distress<br>- Oxygen saturation < 93 % on room air and $PaO_2/FiO_2$ < 250 mmHg<br>- Need no-invasive ventilation: oxygen reservoir or non-rebreathing face mask (oxygen 10-15 L/min) |
| **Critical** | - Positive for SARS-Cov-2 nucleic acid and/or serological test<br>- Admission to intensive-care units<br>- Acute respiratory distress syndrome<br>- Need invasive ventilation<br>- $PaO_2/FiO_2$ < 200 mmHg<br>- With or without one or more additional parameters: need hemodialysis, sepsis, septic shock, and multiorgan dysfunction |

The participants were classified into five clinical groups, established by the symptoms severity, clinical parameters, patient's management and laboratory findings, following the WHO recommendations [1–8]. **Abbreviations:** $FiO_2$ (fraction of inspired oxygen); $PaO_2$ (partial pressure of oxygen).

**Table S2.** Information about r and *p*-values of the correlation matrix shown in Figure 2.

| Comparison | Row | Column | Correlation | *P-value* |
|---|---|---|---|---|
| 1 | Mild | Moderate | -0.231155018 | 0.000332594 |
| 2 | Sat $O_2$ | Moderate | 0.256343743 | 7.28466E-05 |
| 3 | Lymphocyte | Moderate | 0.152904628 | 0.018504855 |
| 4 | BMI | Moderate | -0.077106244 | 0.239009016 |
| 5 | Hypertension | Moderate | -0.004953154 | 0.940185148 |
| 6 | Male | Moderate | -0.001387137 | 0.983052766 |
| 7 | Severe | Moderate | -0.315407459 | 7.15624E-07 |
| 8 | Clinical Score | Moderate | -0.361940265 | 9.56482E-09 |
| 9 | Critical | Moderate | -0.328051133 | 2.37513E-07 |
| 10 | Neutrophil | Moderate | -0.194937891 | 0.002577148 |
| 11 | sTREM-1 | Moderate | -0.305649126 | 1.62032E-06 |
| 12 | Age | Moderate | -0.232030568 | 0.00031521 |
| 13 | IL-10 | Moderate | -0.099067545 | 0.171587141 |
| 14 | IL-6 | Moderate | -0.333358878 | 2.94361E-06 |
| 15 | IL-8 | Moderate | -0.205944097 | 0.005412874 |

| | | | | |
|---|---|---|---|---|
| 16 | IL-1B | Moderate | -0.202705785 | 0.004695755 |
| 17 | IL-12 | Moderate | 0.047893541 | 0.508345685 |
| 18 | TNF | Moderate | 0.001018876 | 0.988779933 |
| 19 | Sat $O_2$ | Mild | 0.493797962 | 8.69017E-16 |
| 20 | Lymphocyte | Mild | 0.473795411 | 1.15153E-14 |
| 21 | BMI | Mild | -0.176923201 | 0.00654393 |
| 22 | Hypertension | Mild | 0.034172795 | 0.604566858 |
| 23 | Male | Mild | -0.187577951 | 0.003752779 |
| 24 | Severe | Mild | -0.319801075 | 4.90581E-07 |
| 25 | Clinical Score | Mild | -0.684083102 | 4.7457E-34 |
| 26 | Critical | Mild | -0.332620874 | 1.57451E-07 |
| 27 | Neutrophil | Mild | -0.486848071 | 1.65221E-15 |
| 28 | sTREM-1 | Mild | -0.473164064 | 1.26229E-14 |
| 29 | Age | Mild | -0.443947281 | 7.24186E-13 |
| 30 | IL-10 | Mild | -0.329159002 | 3.1354E-06 |
| 31 | IL-6 | Mild | -0.524005077 | 1.19155E-14 |
| 32 | IL-8 | Mild | -0.47734009 | 1.08997E-11 |
| 33 | IL-1B | Mild | 0.003209787 | 0.964663496 |
| 34 | IL-12 | Mild | -0.033696836 | 0.641772093 |
| 35 | TNF | Mild | -0.028596493 | 0.693008796 |
| 36 | Lymphocyte | Sat $O_2$ | 0.401200145 | 1.84023E-10 |
| 37 | BMI | Sat $O_2$ | -0.235201534 | 0.000301552 |
| 38 | Hypertension | Sat $O_2$ | -0.064325069 | 0.33249921 |
| 39 | Male | Sat $O_2$ | -0.101669422 | 0.120916986 |
| 40 | Severe | Sat $O_2$ | -0.306087002 | 1.82232E-06 |
| 41 | Clinical Score | Sat $O_2$ | -0.61107148 | 2.39767E-25 |
| 42 | Critical | Sat $O_2$ | -0.321999042 | 4.80411E-07 |
| 43 | Neutrophil | Sat $O_2$ | -0.423331473 | 1.36418E-11 |
| 44 | sTREM-1 | Sat $O_2$ | -0.417465281 | 2.77006E-11 |
| 45 | Age | Sat $O_2$ | -0.387049158 | 8.81104E-10 |
| 46 | IL-10 | Sat $O_2$ | -0.2948569 | 3.81076E-05 |
| 47 | IL-6 | Sat $O_2$ | -0.477628124 | 6.21986E-12 |
| 48 | IL-8 | Sat $O_2$ | -0.416622922 | 7.27459E-09 |
| 49 | IL-1B | Sat $O_2$ | -0.092911818 | 0.202305851 |
| 50 | IL-12 | Sat $O_2$ | -0.008326473 | 0.909224231 |
| 51 | TNF | Sat $O_2$ | -0.120279514 | 0.098329914 |
| 52 | BMI | Lymphocyte | 0.105807313 | 0.105686046 |
| 53 | Hypertension | Lymphocyte | 0.102924798 | 0.117960673 |
| 54 | Male | Lymphocyte | -0.195809682 | 0.002462766 |
| 55 | Severe | Lymphocyte | -0.24467198 | 0.000141946 |
| 56 | Clinical Score | Lymphocyte | -0.504559851 | 1.0349E-16 |
| 57 | Critical | Lymphocyte | -0.284427783 | 8.67676E-06 |
| 58 | Neutrophil | Lymphocyte | -0.244465784 | 0.000143855 |
| 59 | sTREM-1 | Lymphocyte | -0.450771695 | 2.91027E-13 |
| 60 | Age | Lymphocyte | -0.510840741 | 3.72578E-17 |
| 61 | IL-10 | Lymphocyte | -0.4656599 | 1.00449E-11 |
| 62 | IL-6 | Lymphocyte | -0.570302316 | 1.30747E-17 |
| 63 | IL-8 | Lymphocyte | -0.55135014 | 8.75794E-16 |
| 64 | IL-1B | Lymphocyte | -0.055937205 | 0.439722695 |

| | | | | |
|---|---|---|---|---|
| 65 | IL-12 | Lymphocyte | -0.066996635 | 0.35458038 |
| 66 | TNF | Lymphocyte | -0.069797294 | 0.334778798 |
| 67 | Hypertension | BMI | 0.337632848 | 1.54209E-07 |
| 68 | Male | BMI | 0.010023149 | 0.878527219 |
| 69 | Severe | BMI | 0.090491035 | 0.16677444 |
| 70 | Clinical Score | BMI | 0.199649214 | 0.002103156 |
| 71 | Critical | BMI | 0.121363246 | 0.063252208 |
| 72 | Neutrophil | BMI | 0.144400816 | 0.026867836 |
| 73 | sTREM-1 | BMI | 0.080909355 | 0.216558849 |
| 74 | Age | BMI | -0.111379628 | 0.088452531 |
| 75 | IL-10 | BMI | -0.064540076 | 0.375061815 |
| 76 | IL-6 | BMI | 0.10571597 | 0.149870463 |
| 77 | IL-8 | BMI | 0.079989279 | 0.285789589 |
| 78 | IL-1B | BMI | -0.048881404 | 0.500756237 |
| 79 | IL-12 | BMI | -0.049949198 | 0.491436097 |
| 80 | TNF | BMI | -0.003880337 | 0.957399957 |
| 81 | Male | Hypertension | 0.008049538 | 0.902941275 |
| 82 | Severe | Hypertension | 0.027496362 | 0.676949976 |
| 83 | Clinical Score | Hypertension | -0.02087052 | 0.751844854 |
| 84 | Critical | Hypertension | -0.052086696 | 0.429754465 |
| 85 | Neutrophil | Hypertension | -0.039892941 | 0.545451521 |
| 86 | sTREM-1 | Hypertension | -0.024131327 | 0.714644644 |
| 87 | Age | Hypertension | -0.048797374 | 0.459490751 |
| 88 | IL-10 | Hypertension | -0.176737196 | 0.015255029 |
| 89 | IL-6 | Hypertension | -0.076601215 | 0.301366345 |
| 90 | IL-8 | Hypertension | -0.155970027 | 0.037078899 |
| 91 | IL-1B | Hypertension | -0.088646282 | 0.225133769 |
| 92 | IL-12 | Hypertension | -0.070127011 | 0.337623046 |
| 93 | TNF | Hypertension | 0.089498929 | 0.220686885 |
| 94 | Severe | Male | 0.036825251 | 0.572678206 |
| 95 | Clinical Score | Male | 0.191474889 | 0.003080732 |
| 96 | Critical | Male | 0.12249276 | 0.05971684 |
| 97 | Neutrophil | Male | 0.031451675 | 0.629982399 |
| 98 | sTREM-1 | Male | 0.163859007 | 0.011525823 |
| 99 | Age | Male | 0.104158239 | 0.109738178 |
| 100 | IL-10 | Male | 0.247127239 | 0.000548883 |
| 101 | IL-6 | Male | 0.119514701 | 0.102335595 |
| 102 | IL-8 | Male | 0.173228274 | 0.019697498 |
| 103 | IL-1B | Male | -0.140872767 | 0.050688253 |
| 104 | IL-12 | Male | -0.051524688 | 0.476695911 |
| 105 | TNF | Male | -0.075172413 | 0.29879757 |
| 106 | Clinical Score | Severe | 0.06320037 | 0.332653938 |
| 107 | Critical | Severe | -0.453856055 | 1.9146E-13 |
| 108 | Neutrophil | Severe | 0.091391754 | 0.160779086 |
| 109 | sTREM-1 | Severe | 0.109629133 | 0.092205835 |
| 110 | Age | Severe | 0.223739234 | 0.000519861 |
| 111 | IL-10 | Severe | 0.133051116 | 0.065803523 |
| 112 | IL-6 | Severe | 0.339610294 | 1.86137E-06 |
| 113 | IL-8 | Severe | 0.18170797 | 0.014360805 |

| | | | | |
|---|---|---|---|---|
| 114 | IL-1B | Severe | -0.087249961 | 0.227607984 |
| 115 | IL-12 | Severe | -0.047658186 | 0.510434151 |
| 116 | TNF | Severe | -0.166640725 | 0.020545856 |
| 117 | Critical | Clinical Score | 0.814088698 | 2.18852E-57 |
| 118 | Neutrophil | Clinical Score | 0.60170919 | 9.89693E-25 |
| 119 | sTREM-1 | Clinical Score | 0.670648205 | 2.51929E-32 |
| 120 | Age | Clinical Score | 0.527521057 | 2.22592E-18 |
| 121 | IL-10 | Clinical Score | 0.39350729 | 1.63783E-08 |
| 122 | IL-6 | Clinical Score | 0.706591772 | 9.50877E-30 |
| 123 | IL-8 | Clinical Score | 0.611328472 | 6.24207E-20 |
| 124 | IL-1B | Clinical Score | 0.193142907 | 0.007119883 |
| 125 | IL-12 | Clinical Score | 0.028906508 | 0.689851391 |
| 126 | TNF | Clinical Score | 0.13516282 | 0.060909273 |
| 127 | Neutrophil | Critical | 0.48144692 | 3.72683E-15 |
| 128 | sTREM-1 | Critical | 0.544209066 | 1.13204E-19 |
| 129 | Age | Critical | 0.345890241 | 4.59209E-08 |
| 130 | IL-10 | Critical | 0.247996578 | 0.000523876 |
| 131 | IL-6 | Critical | 0.435159773 | 4.35884E-10 |
| 132 | IL-8 | Critical | 0.418124958 | 4.71667E-09 |
| 133 | IL-1B | Critical | 0.27583581 | 0.000103287 |
| 134 | IL-12 | Critical | 0.036207063 | 0.617144789 |
| 135 | TNF | Critical | 0.200079903 | 0.005273927 |
| 136 | sTREM-1 | Neutrophil | 0.594134952 | 5.25175E-24 |
| 137 | Age | Neutrophil | 0.337763026 | 9.83417E-08 |
| 138 | IL-10 | Neutrophil | 0.251205264 | 0.000440416 |
| 139 | IL-6 | Neutrophil | 0.409908655 | 5.16878E-09 |
| 140 | IL-8 | Neutrophil | 0.374089043 | 2.1304E-07 |
| 141 | IL-1B | Neutrophil | 0.115544468 | 0.109569412 |
| 142 | IL-12 | Neutrophil | 0.002980357 | 0.967187816 |
| 143 | TNF | Neutrophil | 0.159421183 | 0.026791288 |
| 144 | Age | sTREM-1 | 0.488857505 | 1.21629E-15 |
| 145 | IL-10 | sTREM-1 | 0.347496792 | 7.87725E-07 |
| 146 | IL-6 | sTREM-1 | 0.565137436 | 2.94966E-17 |
| 147 | IL-8 | sTREM-1 | 0.579913342 | 1.18423E-17 |
| 148 | IL-1B | sTREM-1 | 0.148895405 | 0.03877017 |
| 149 | IL-12 | sTREM-1 | 0.070661441 | 0.328814567 |
| 150 | TNF | sTREM-1 | 0.107860689 | 0.13541786 |
| 151 | IL-10 | Age | 0.362968733 | 2.28949E-07 |
| 152 | IL-6 | Age | 0.560872117 | 5.71432E-17 |
| 153 | IL-8 | Age | 0.537764866 | 5.89503E-15 |
| 154 | IL-1B | Age | 0.111105111 | 0.123984768 |
| 155 | IL-12 | Age | 0.053394695 | 0.460824155 |
| 156 | TNF | Age | 0.083246073 | 0.249741408 |
| 157 | IL-6 | IL-10 | 0.595828669 | 1.88665E-19 |
| 158 | IL-8 | IL-10 | 0.691523071 | 4.45477E-27 |
| 159 | IL-1B | IL-10 | 0.084361802 | 0.244669961 |
| 160 | IL-12 | IL-10 | 0.066528425 | 0.359225709 |
| 161 | TNF | IL-10 | 0.029557013 | 0.684031098 |
| 162 | IL-8 | IL-6 | 0.75569478 | 2.30662E-34 |

| | | | | |
|---|---|---|---|---|
| 163 | IL-1B | IL-6 | 0.139639406 | 0.055973197 |
| 164 | IL-12 | IL-6 | 0.065061294 | 0.375043236 |
| 165 | TNF | IL-6 | 0.002706864 | 0.9705909 |
| 166 | IL-1B | IL-8 | 0.042398459 | 0.57090717 |
| 167 | IL-12 | IL-8 | 0.004694633 | 0.949987402 |
| 168 | TNF | IL-8 | -0.008794129 | 0.906467413 |
| 169 | IL-12 | IL-1B | 0.268492309 | 0.000159788 |
| 170 | TNF | IL-1B | 0.328036453 | 3.20628E-06 |
| 171 | TNF | IL-12 | 0.377835832 | 6.05668E-08 |

**Table S3.** Multivariate Regression Model* for Death in the Study Population.

| Variable | IRR** (95% Confidence Interval) | *p*-value |
|---|---|---|
| **Sex (Male)** | 1.05 (0.75 – 1.47) | 0.771 |
| **Age over 60 years** | 2.05 (1.31 – 3.22) | 0.002 |
| **Days of Disease** | 1.01 (0.90 – 1.04) | 0.174 |
| **Comorbidities** | 1.11 (0.90 – 1.24) | 0.069 |
| **Severity Score** | 1.65 (1.23 – 2.22) | 0.001 |
| **Neutrophils-Lymphocytes Ratio** | 0.99 (0.90 – 1.10) | 0.612 |
| **High TREM-1** | 1.05 (0.75 – 1.47) | 0.003 |

*Model *p*-value < 0.0001, Deviance Goodness-of-fit 65.35, *p* = 1.00, Pearson Goodness-of-fit 59.65, *p* = 1.00.
**Incidence Rate Ratio

**Table S4.** Hospital support, supportive therapies, and medications of patients moderate, severe, and critical infected with SARS-CoV-2.

| During Hospital Stay | Moderate/Severe/critical N=33 |
|---|---|
| **Hospital support, No. (%)** | |
| Infirmary | 20 (60.6) |
| Intensive care unit (ICU) | 13 (39.4) |
| **Hospitalization data, No.** | |
| Hospitalization days, median (IQR) | 11 (3-30) |
| Number of days since symptom onset (IQR) | 6 (3-11) |
| **Respiratory support received (%)** | |
| Nasal-cannula oxygen | 13 (39.4) |
| Oxygen mask | 11(33.3) |
| Invasive mechanical ventilation | 9 (27.3) |
| Oxygen Saturation median (IQR) | 89 (54-99) |
| **Medications** | |
| Glucocorticoid | 31 (93.9) |
| Antibiotics | 33 (100) |
| Oseltamivir | 19 (57.6) |
| Chloroquine/Hydroxychloroquine | 10 (30.3) |
| Anticoagulants | 11 (33.3) |
| **Death** | 24 (72.7) |

**Abbreviation:** IQR, interquartile range, Data are median (IQR), n (%), or n/N.

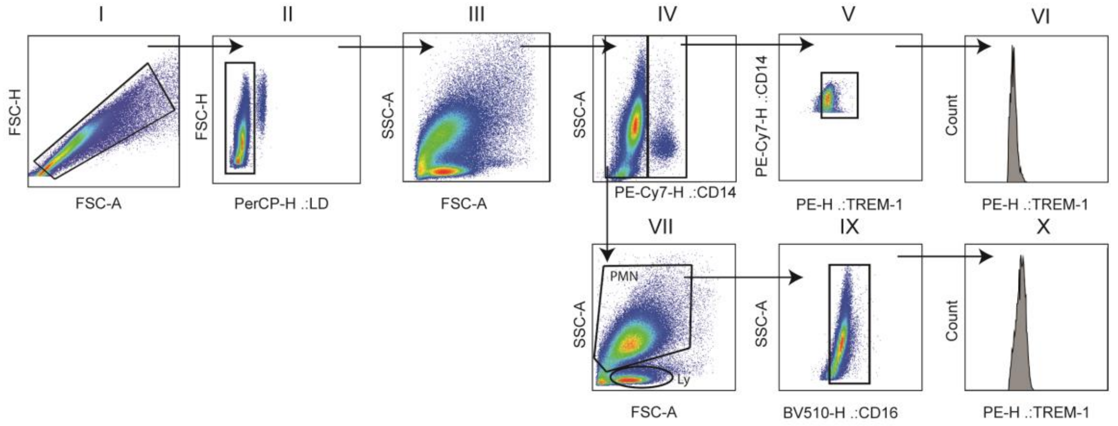


**Figure S1.** Gating strategy used for flow cytometry analysis of peripheral blood leukocytes. Dot plots shown a representative gating strategy for the analysis of (I) singlet gating based FSC-H/FSC-A, (II) FSC-H/FVS-620 (viable cells), (III) SSC-A/FSC-A, followed by (IV) SSC-A/ leukocytes gated according to their side scatter and CD14 (PE-Cy7) antibody staining patterns, (V) CD14 (PE-Cy7) antibody staining patterns versus TREM-1 (PE) antibody staining patterns, with subsequent (VI) TREM-1 (PE) mean fluorescence intensity (MFI); (VII) light scatter flow cytometry profile for cells based on forward scatter (FSC-A) related to size, and side scatters (SSC-A) related to granularity; (IX) gated according to their side scatter and CD16 (BV510) antibody staining patterns, with subsequent (X) TREM-1 mean fluorescence intensity (MFI).

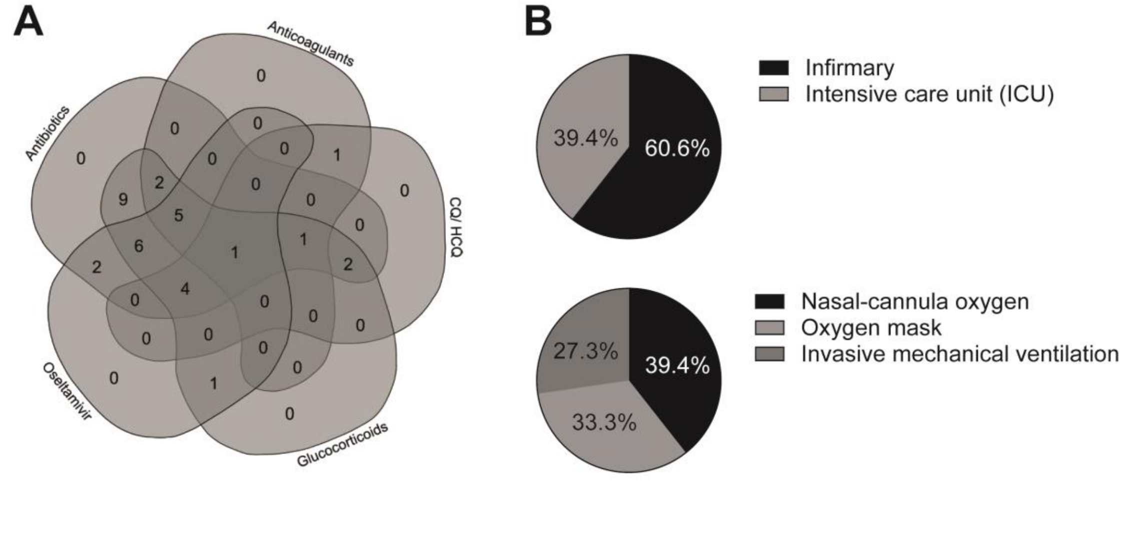


**Figure S2.** Venn diagram of the pharmacological treatment of patients with COVID-19 and hospital/ respiratory support during hospitalization. (A) Venn diagram showed all pharmacological treatment relations between Glucocorticoid, Antibiotics, Oseltamivir, Chloroquine/Hydroxychloroquine and Anticoagulants of different sets of patients with COVID-19 in hospital care. (B) Demonstrative percentages of patients in distinctive hospitals and respiratory support during hospitalization.

**References**


1. Ye, G.; Pan, Z.; Pan, Y.; Deng, Q.; Chen, L.; Li, J.; Li, Y.; Wang, X. Clinical Characteristics of Severe Acute Respiratory Syndrome Coronavirus 2 Reactivation. *Journal of Infection* **2020**, *80*, doi:10.1016/j.jinf.2020.03.001.

2. Xu, X.W.; Wu, X.X.; Jiang, X.G.; Xu, K.J.; Ying, L.J.; Ma, C.L.; Li, S.B.; Wang, H.Y.; Zhang, S.; Gao, H.N.; et al. Clinical Findings in a Group of Patients Infected with the 2019 Novel Coronavirus (SARS-Cov-2) Outside of Wuhan, China: Retrospective Case Series. *The BMJ* **2020**, *368*, doi:10.1136/bmj.m606.

3. Grasselli, G.; Zangrillo, A.; Zanella, A.; Antonelli, M.; Cabrini, L.; Castelli, A.; Cereda, D.; Coluccello, A.; Foti, G.; Fumagalli, R.; et al. Baseline Characteristics and Outcomes of 1591 Patients Infected with SARS-CoV-2 Admitted to ICUs of the Lombardy Region, Italy. *JAMA - Journal of the American Medical Association* **2020**, *323*, 1574–1581, doi:10.1001/jama.2020.5394.

4. Marshall, J.C.; Murthy, S.; Diaz, J.; Adhikari, N.; Angus, D.C.; Arabi, Y.M.; Baillie, K.; Bauer, M.; Berry, S.; Blackwood, B.; et al. A Minimal Common Outcome Measure Set for COVID-19 Clinical Research. *The Lancet Infectious Diseases* 2020, *20*.

5. Office, W.H.O.E.M.R. Updated Clinical Management Guideline for COVID-19. *Weekly Epidemiology Monitor* 2020, *13*.

6. Wei, P.-F. Diagnosis and Treatment Protocol for Novel Coronavirus Pneumonia (Trial Version 7). *Chinese Medical Journal* **2020**, *133*, 1087–1095, doi:10.1097/CM9.0000000000000819.

7. Wan, S.; Xiang, Y.; Fang, W.; Zheng, Y.; Li, B.; Hu, Y.; Lang, C.; Huang, D.; Sun, Q.; Xiong, Y.; et al. Clinical Features and Treatment of COVID-19 Patients in Northeast Chongqing. *Journal of Medical Virology* **2020**, *92*, 797–806, doi:10.1002/jmv.25783.

8. Hadjadj, J.; Yatim, N.; Barnabei, L.; Corneau, A.; Boussier, J.; Smith, N.; Péré, H.; Charbit, B.; Bondet, V.; Chenevier-Gobeaux, C.; et al. Impaired Type I Interferon Activity and Inflammatory Responses in Severe COVID-19 Patients. *Science* **2020**, *369*, doi:10.1126/science.abc6027.
